# Urban and sub-urban disparities in health outcomes among patients with COVID-19; a cross-sectional study of 234 418 patients in Iran

**DOI:** 10.1186/s12889-022-13290-x

**Published:** 2022-05-10

**Authors:** Mohammad-Reza Sohrabi, Rozhin Amin, Ali Maher, Khatereh Hannani, Hossein Alimohammadi, Ali-Reza Zali

**Affiliations:** 1 Community Medicine Department, School of Medicine, Shahid Beheshti University of Medical Sciences, Tehran, Iran; 2 Social Determinants of Health Research Center, Shahid Beheshti University of Medical Sciences, Tehran, Iran; 3 School of Management and Medical Education, Shahid Beheshti University of Medical Sciences, Tehran, Iran; 4 Statistics & Information Technology Management, Shahid Beheshti University of Medical Sciences, Tehran, Iran; 5 Emergency Department, Imam Hossein Hospital, Shahid Beheshti University of Medical Sciences, Tehran, Iran; 6 Functional Neurosurgery Research Center, Shahid Beheshti University of Medical Sciences, Tehran, Iran

**Keywords:** Covid-19, Determinants, Disparities, Health outcome, Iran, Pandemic, SARS-CoV-2, Spatial, Sub-urban, Urban

## Abstract

**Background:**

It remains crucial to understand socio-demographic determinants of COVID-19 infection to improve access to care and recovery rates from the disease. This study aimed to investigate the urban and sub-urban disparities associated with COVID-19 in patients visiting healthcare facilities in the province of Tehran, Iran.

**Methods:**

Data from 234 418 patients who were diagnosed with COVID-19 infection from March 2020 to March 2021 in the province of Tehran were used in this analysis. Descriptive statistics were used to describe the characteristics of the study population. Chi-Squared test was applied to examine the association of study variables with residing area. Independent samples t-test was performed to compare mean age of patients in urban and sub-urban areas. Multiple Logistic Regression model was applied to examine the association of study variables with disease outcome.

**Results:**

Overall, most patients resided in the urban settings (73%). Mean age of patients was significantly lower in sub-urban areas compared to their counterparts in urban settings (49 ± 23.1 years versus 53 ± 21.1 years, *P* < 0.001). Positive PCR test results were more common in urban areas (48.5% versus 41.3%, *P* < 0.001). Yet, sub-urban settings had higher rates of positive chest CT scan reports (62.8% versus 53.4%, *P* < 0.001). After accounting for age and sex covariates, residing in urban areas was associated with higher likelihood of being admitted to an ICU (OR = 1.27, CI: 1.240-1.305). Yet, a greater vulnerability to fatal outcome of COVID-19 infection was shown in patients living in sub-urban areas (OR = 1.13, CI: 1.105-1.175).

**Conclusions:**

This study revealed a clear disparity in the health outcome of patients infected with COVID-19 between urban and sub-urban areas.

## Background

The Severe Acute Respiratory Syndrome Coronavirus 2 (SARS-CoV-2), which was first detected in China, has now affected more than 200 countries and territories worldwide. The human virus emerged in the late 2019, causing respiratory illness, yet, gastrointestinal, cardiological, dermatological, and neurological symptoms were later added to the wide range of events that the virus could present with [1]. In early 2020, the World Health Organization (WHO) officially named the disease as COVID-19 and declared it as a “Public Health Emergency of International Concern” [2, 3]. To date, about 340 million confirmed cases with over 5.5 million casualties have been reported globally (January 2022) [4].

Iran is among the counties worst hit by SARS-CoV-2 virus. The country was first confirmed to have been affected on February 19, 2020, when two COVID-19 related deaths were reported in the city of Qom, north-central Iran. In early March, almost all regions of the country had reported coronavirus infected cases [5]. By the time of writing this paper, in January 2022, over six million confirmed cases and over 132 000 confirmed mortalities have been officially recorded in Iran [6]. However, rates continue to grow as the COVID-19 pandemic unfolds and more people become affected by the disease [7].

Studies have shown considerable variations in the incidence and consequences associated with COVID-19 among communities. It is widely believed that different socio-demographic characteristics of populations could greatly contribute to the observed disparities. While studies on socio-demographic factors determining people’s health during COVID-19 pandemic are sparse, available data suggest that factors such as sex, age, health literacy, income, and residing area influence the vulnerability of societies to the infection [8, 9]. Studies conducted in Iran have revealed that older age, male sex, and low literacy are associated with poor prognosis related to COVID-19 health outcome [10, 11]. Moreover, they have indicated that disease spread, and COVID-19 fatality rate were spatially correlated within the province of Tehran. Throughout the epidemic, higher case numbers and mortality rates were reported from central and south parts of the province where mainly people from lower socio-economic status reside compared to other parts in the province [11, 12]. Discrepancies in the health outcome of patients diagnosed with COVID-19 in different residing settings have also been shown in the literature [13, 14]. Yet, to effectively support decision making about COVID-19 containment strategies, understanding the magnitude and how area of residence could impact the health outcome of the pandemic remains crucial in countries where urban-suburban disparities in access to healthcare exits [15]. Given the paucity of research on the above-mentioned topics, this study aimed to investigate the urban and sub-urban disparities associated with COVID-19 health outcome in patients diagnosed with the disease in the province of Tehran.

## Methods

A cross-sectional study was performed using data from the registry of Coronavirus Control Operations Headquarter in Tehran. Tehran’s COVID-19 registry compiles information on all clinically suspected patients of COVID-19 who had visited the designated healthcare facilities across the province, where they were assessed by either chest CT or molecular polymerase chain reaction (PCR) test. Results were available to the patients through qualified healthcare providers responsible for informing the patient about the result, doing the follow-ups, and filing the records. Tehran is a province in north-central Iran, which is home to the city of Tehran, Iran’s capital. It is the nation’s most populous province, with 13 267 637 inhabitants recorded in the 2016 national census. Over 70% (*n* = 8 693 706) of its population live in urban areas [16]. All patients who had seen the COVID-19 designated healthcare facilities from March 2020 to March 2021 in the province of Tehran and were diagnosed with the infection were included in the study. Overall, data on socio-demographic characteristics, test results, and the health outcome of 234 418 patients were extracted from the COVID-19 registry.

### Variables

Patients were characterized by age, sex, residing area, test result, chest CT, intensive care unit (ICU) admission, and the disease outcome. For ease of interpretation, all variables were categorized. Patients were split into 5 age groups: < 17.9, 18-39.9, 40-59.9, 60-79.9, and > 80 as the oldest age group. Sex was defined as being a man or woman. The place of residence and home address was asked from the patients at the time of admission. The patients were later categorized into residences of either urban or sub-urban areas based on their responses and according to municipality classification provided by Statistics Center of Iran [17]. Test results were classified based on the National Coronavirus Control Operations Headquarter protocol as negative, positive, or inconclusive. Chest CT was divided into reports with positive COVID-19 related findings and reports which were negative for COVID-19 related findings. The disease outcome (main outcome) was categorized as recovered, under treatment, or deceased. ICU admission (secondary outcome) was documented as yes or no.

### Statistical analysis

Descriptive statistics (absolute and relative frequencies) were used to describe the characteristics of the study population. The Chi-Squared test was applied to examine the association of study variables with residing area. Independent samples t-test was performed to compare mean age of patients in urban and sub-urban areas. Since age and sex were key determinants that may confound the relationship between residing area and the health outcome, Multiple Logistic Regression model was fitted to determine the association of ICU admission and disease outcome with residing area while adjusted for age and sex co-variates. To this purpose, dependent variables were defined as ICU admission which was classified as either admitted or not admitted and disease outcome which was dichotomized into being alive or deceased. The estimates were analysed by using IBM SPSS Statistics, version 26 (IBM Corp., Armonk, N.Y., USA), considering a significance level of α < 0.05.

## Results

Data on 234 418 COVID-19 patients (54 % men, 46 % women) were analysed. About a third aged 40 years and younger (28%, *n* = 62 214), from which 7% (*n* = 14 916) were among the children under 18 years of age. The proportion of children aged 18 years and younger who were diagnosed with COVID-19 was higher in sub-urban communities compared to urban areas, 11% versus 5%. Overall, most patients resided in the urban settings (73%, *n* = 165 543). PCR test results were positive for SARS-CoV-2 in less than half of the patients in both settlements. However, positive test results were more common in urban areas. Among all, almost 60% had COVID-19 related findings in their Chest CT scan, yet sub-urban settings had higher rates of positive reports. The characteristics of the study population is summarized in Table 1.

**Table 1:** The Characteristics of the Study Population

**Characteristics**		**urban**		**suburban**		**Overall**		***P* value**
		n	%	n	%	n	%	
**Age**	< 17.9	8577	5.2	6339	10.6	14916	6.6	< 0.001
	18-39.9	34317	20.7	12981	21.6	47298	21.0	
	40-59.9	51224	30.9	18414	30.7	69638	30.9	
	60-79.9	52732	31.9	17062	28.4	69794	30.9	
	> 80	18693	11.3	5178	8.6	23871	10.6	
**Sex**	Female	75007	45.3	28383	47.3	103390	45.8	< 0.001
	Male	90536	54.7	31591	52.7	122127	54.2	
**Test result**	Negative	51200	43.6	22668	50.1	73868	45.4	< 0.001
	Inconclusive	9322	7.8	3918	8.2	13240	8.0	
	Positive	57031	48.5	18686	41.3	75717	46.5	
**Chest CT findings**	Negative	77218	46.6	22324	37.2	99542	44.1	< 0.001
	Positive	88325	53.4	37650	62.8	125975	55.9	

*n* number of patients in each category; %, percentage of patients in each category

Results from the independent samples t-test comparing the mean age of patients between the two groups revealed a significant difference in mean age of patients between urban residents (Mean = 53, SD = 21.1) and sub-urban residents (Mean = 49, SD = 23.1); t _(98392)_ = 41.6, *p* < 0.001.

Table 2 demonstrates the distribution of the COVID-19 health outcome in the study population. In total, about twenty percent of the study population were admitted to an ICU. The ICU admission rate was higher in urban areas compared to sub-urban communities. The overall mortality rate related to COVID-19 was estimated at 11.6%. The rates were similar in both urban and sub-urban settings in unadjusted comparison. However, recovery rate was higher in urban areas. In both settings, nearly one in three patients admitted to an ICU died in the hospital. The death rate in patients who were admitted to an ICU was 33.8% in urban areas and 33.5% in sub-urban areas. In both areas, higher death rates were seen in patients over 80 years old (30.1% in urban areas and 28.6 % in sub-urban areas), followed by patients in 60 to 79.9 years’ age group (17.2% in urban areas and 19.5 in sub-urban areas). Greater proportion of men patients died with the disease when compared to women (12.8% versus 10.2 in urban areas, 12.4% versus 10.7 in sub-urban areas).

**Table 2:** The Distribution of the COVID-19 Health Outcome

**Characteristics**		**Urban**		**Sub-urban**		**Total**		***P* value**
		n	%	n	%	n	%	
**ICU admission**	No	131879	79.9	50408	84.0	182287	80.8	< 0.001
	Yes	33664	20.3	9566	16.0	43230	19.2	
**Outcome**	Recovered	143962	87.0	51177	85.3	195139	86.5	< 0.001
	Under treatment	2322	1.4	1854	3.1	4176	1.9	
	Deceased	19259	11.6	6943	11.6	26202	11.6	

*n* number of patients in each category; %, percentage of patients in each category

In the prognostic model of ICU admission, adults aged 18-39 years had lower risk for being admitted to an ICU compared to children under the age of 18 who were considered as reference group in the analysis. An insignificant difference was observed for the probability of being admitted to an ICU between children under the age of 18 and adults aged 40 to 49. However, the odds of requiring intensive care had significantly increased from the age of 60 years. Men were more likely to require critical care than women. Living in urban areas were associated with greater risk of ICU hospitalisation (Table 3).

**Table 3:** Logistic Regression Model of Independent Variables Associated with COVID-19 Related ICU admission

**variable**	**aOR**	**95% Confidence Interval**		***p*-value**
		**Lower**	**Upper**	
**Age group**				
< 17.9	1			
18-39.9	0.55	0.528	0.590	<0.001
40-59.9	0.97	0.928	1.027	0.34
60-79.9	1.98	1.890	2.084	<0.001
> 80	3.13	2.975	3.30	<0.001
**Sex**				
Female	1			
Male	1.12	1.102	1.151	<0.001
**Residing area**				
Sub-urban	1			
Urban	1.27	1.240	1.305	<0.001
**Constant**	0.13			<0.001

*aOR* adjusted Odds Ratio

Results from the multivariate logistic regression analysis of COVID-19 related death determinants are provided in Table 4. The odds of death from COVID-19 infection increased with age starting from the age of 40 years. Male patients were more likely to die with the disease than female patients. After accounting for age and sex covariates, residing in sub-urban areas was associated with higher likelihood of death from the COVID-19.

**Table 4:** Logistic Regression Model of Independent Variables Associated with COVID-19 Related Death

**variable**	**aOR**	**95% Confidence Interval**		***p*-value**
		**Lower**	**Upper**	
**Age group**				
< 17.9	1			
18-39.9	0.64	0.584	0.716	<0.001
40-59.9	1.98	1.816	2.170	<0.001
60-79.9	5.67	5.204	6.186	<0.001
> 80	11.17	10.222	12.209	<0.001
**Sex**				
Female	1			
Male	1.31	1.275	1.347	<0.001
**Residing area**				
Urban	1			
Sub-urban	1.13	1.105	1.175	<0.001
**Constant**	0.03			<0.001

*aOR* adjusted Odds Ratio

## Discussion

This study indicated that the type of residing area could impact patients in terms of COVID-19 incidence and health outcome and might be considered as an important prognostic social determinant. Results showed that the mean age of men and women with COVID-19 infection were significantly lower in sub-urban areas compared to their counterparts in urban settings. After accounting for age and sex covariates, residing in urban areas was associated with higher likelihood of being admitted to an ICU. Yet, a greater vulnerability to fatal outcome of COVID-19 infection was shown in patients living in sub-urban areas of the province after controlling for age and sex.

The mortality rate in the study population was 11.6% in both urban and sub-urban areas. However, after controlling the confounding effect of sex and age in the multivariate analysis, a clear disparity in the probability of death due to COVID-19 was observed between urban and sub-urban areas. Patients residing in sub-urban areas were more likely to die of the disease compared to those living in urban settings. The geographic disparities in the impact of COVID-19 have also been found in multiple studies from United States [18–21]. This finding could partly be explained by the spatial disparities in the availability and accessibility of the health-care resources. Urban areas have superior healthcare system in terms of treatment options and are more prepared for critically ill patients. Whereas there are fewer hospitals in sub-urban areas and less access to medical specialists [22]. Therefore, outbreaks in those areas could rapidly overwhelm the healthcare facilities and lead to higher mortality rates compared to urban settings. However, a more important explanation for the higher vulnerability of sub-urban population to serious health outcome of the disease, is the lower socio-economic status of the people in those areas with a grater prevalence of malnutrition and comorbidities, which adversely affects the course of the disease [23]. Moreover, many in sub-urban areas are low-wage day workers, who are reluctant to get tested in fear of losing work or the day’s pay [24]. This could result in delayed testing and worst condition of these patients by the time of admission which ends up in more severe health outcomes in those areas. Though the income level is generally lower in sub-urban areas compared to urban settings, the effect of income discrepancies on the health outcome of patients may not be significant [22]. It is mainly due to the fact that across the province the majority of patients diagnosed with COVID-19 are treated in public healthcare settings where the costs are mainly covered by government with minimal amounts of out-of-pocket payments. Therefore, affordability of treatment services was considered to have minimal roll in the disparities observed between urban and sub-urban areas.

The ICU admission rate was 20% for urban areas versus 16% in sub-urban settings. The spatial differences in ICU admission rates have also been reported in other studies [25, 26]. The lower rates in sub-urban areas observed in this study, may be attributed to the lower mean age of patients who had visited the healthcare facilities in these settings during the epidemic. Nevertheless, this finding should be interpreted with caution as critically ill patients could have been treated outside the intensive care unit due to various reasons such as ICU beds unavailability, age, and existing comorbidities.

In this study, seventy percent of the patients were reported from the urban areas versus 30% from sub-urban settings, which is consistent with expectations, since two-thirds of the province’s population live in urban areas. Yet, higher population density in urban areas could also facilitate human contacts and contribute to greater infected cases in those settings. Another possible explanation for the lower reported cases from sub-urban areas is the lower testing rates in those areas. The geographic disparities in COVID-19 testing rates have also been observed in other countries and could be attributed to a wide range of barriers including less available testing sites, transportation limitations, and lack of information [25, 27, 28].

PCR test results were positive for SARS-CoV-2 in less than half of the patients in both settlements, with urban areas showing higher rate for positive results. Since during the study period, PCR testing was limited to the patients who were clinically or para-clinically highly suspicious for COVID-19 in both areas, it is probable that the observed difference between the two areas is related to the existing issues with test accuracy. The accuracy of the PCR testing in clinical setting is dependent on the site and sampling quality. The sensitivity of the test was reported as 63% for nasal swabs, and 32% for throat swabs. It also varies depending on the stage of the disease and the degree of viral multiplication. A 38% false negative rate was reported for testing on the day of symptom onset, which tends to decrease to 20% eight days post symptom onset, and then gradually increases to 66% by day 21 [29, 30]. Given the fact that sub-urban settings had higher rate of positive chest CT reports by the time of admission, it is presumable that lower rate of positive PCR test results in these areas is due to the delayed testing of patients in sub-urban areas [31, 32].

The mean age of patients with COVID-19 infection was significantly lower in sub-urban areas compared to their counterparts in urban settings. The sub-urban areas in the province of Tehran are inhabited by a disproportionate number of young adults who had migrated from smaller cities in search of job. They are mostly essential workers who need to frequently travel to the core city for work reasons. Therefore, consistent application of prevention measures might be difficult or impossible. The higher proportion of children aged 18 years and younger who were diagnosed with COVID-19 in sub-urban areas could also reflect the higher number of younger families residing in these areas [33]. However, since the data consists symptomatic patients, this finding could also point to the higher prevalence of malnutrition and untreated comorbidities in the children in the lower income sub-urban settings. The conditions that put children at risk for developing more severe illness [34, 35].

The current study was limited by the fact that the testing was relied on secondary analysis of existing data, therefore assessment of factors associated with COVID-19 incidence, disease duration, and health outcome were limited to available information and the current logistic regression model could face several residual confounding due to multiple variables associated with COVID-19 and death. Generally, a combination of population and individual level factors contribute to the geographic disparities of a disease impact, but due to lack of data on patients’ income, education, and occupation complete assessment of the disparities at the individual level was restricted. Overall, it should be noted that asymptomatic patients and those with mild symptoms who have not visited the healthcare facilities during the study period were not included in the study population. Therefore, the observed rates might not reflect the mortality rates in general population. In addition, inherent to the nature of the cross-sectional studies, causal inferences should be done with cautious. However, the strength of this work lies in the use of a large multicentre sample of COVID-19 patients, including all ages and both sexes from urban and sub-urban areas of the province which have greatly contributed to reliable extension of inferences to the target population. Furthermore, due to the uniform data collection procedure and existence of a centralized healthcare information system in the province, a high-quality data was made available to the researchers.

## Conclusions

Residing in sub-urban areas was associated with lower likelihood of being admitted to an ICU, but greater vulnerability to fatal outcome of COVID-19 infection. Findings about correlates of the disease, could aid decision makers to be proactively cautious about vulnerable populations and inform their efforts in addressing the uneven spatial distribution of the disease burden.

## Data Availability

The data that support the findings of this study are available from the Coronavirus Control Operations Headquarter in the province of Tehran, but restrictions apply to the availability of these data, which were used under license for the current study, and so are not publicly available. Data are however available from the corresponding author, Ali-Reza Zali, upon reasonable request and with permission of the Coronavirus Control Operations Headquarter in the province of Tehran.

## Abbreviations

SARS-CoV-2: Severe Acute Respiratory Syndrome Coronavirus 2
WHO: World Health Organization
PCR: Polymerase Chain Reaction
ICU: Intensive Care Unit
